# A Randomized Trial of Grant Writing Coaching Groups: Qualitative Interviews Revealing Key Elements of Intervention Efficacy

**DOI:** 10.64898/2026.09.17.752282

**Published:** 2026-09-21

**Authors:** Julia Mendes, Christine Wood, Remi Jones, Jeffrey Engler, Anne Marie Weber-Main, Harlan Jones, Melanie Steiner, Kola Okuyemi, Richard McGee

## Abstract

**Background:**

Training in grant proposal writing is an essential component of professional development for academic scientists in the biomedical and behavioral sciences. Despite the expansion of inter- and intra-institutional grant writing coaching groups as an approach to honing these skills, specific features that enhance or limit coaching group effectiveness have not been rigorously studied.

**Methods:**

Qualitative interviews were conducted with a subset of early-career investigators (n=204 and coaches (n=36) engaged in a national, U.S. based, group-randomized trial of grant writing coaching groups to test the effects of two variables on submission and funding of national-level proposals: (1) coaching duration (regular/extended dose) and (2) mode of engaging a scientific advisor (someone with content-aligned expertise) in the coaching process. This report focuses on interviews conducted upon completion of the regular coaching dose – 5 months of biweekly, group-based coaching sessions to support active proposal writing. Interviews were designed to identify which coaching group elements were perceived to be the most critical. Transcribed interviews were analyzed using deductive (participants) or open (coaches) coding to identify themes.

**Results:**

Triangulation of results from participant and coach interviews showed strong concordance that coaches, peers, and scientific advisors all played key roles in supporting intervention efficacy. Sufficient alignment of scientific fields and/or methodologies among group members was important, although breadth of perspectives was also valued. Other critical group features were skilled and well-organized coaches, detailed feedback, peer-to-peer support (technical and psychosocial), and clear expectations for group functioning. Factors attenuating impact included variation in participants’ engagement and “readiness to write,” within-group mismatches of expertise or grant mechanisms, and limited research support at some participants’ home institutions.

**Conclusion:**

This study identified key elements of successful grant writing coaching groups and potential barriers to their effectiveness, while yielding insights about tailoring this approach for individuals at different stages of proposal development.

## Full Text Availability

The license terms selected by the author(s) for this preprint version do not permit archiving in PMC. The full text is available from the preprint server.

